# Smartphone-based drug testing in the hands of patients with substance-use disorder—a usability study

**DOI:** 10.3389/fdgth.2024.1394322

**Published:** 2024-09-02

**Authors:** Johan Månflod, Tove Gumbel, Maria Winkvist, Markku D. Hämäläinen, Karl Andersson

**Affiliations:** ^1^ Region Uppsala, Needle Exchange Programme, Uppsala, Sweden; ^2^ Kontigo Care AB, Uppsala, Sweden; ^3^ Skillsta Teknik Design och Kvalitet AB, Vänge, Sweden; ^4^ IGP, Uppsala University, Uppsala, Sweden

**Keywords:** addiction, eHealth (mobile health), substance-use disorders, eye-scanning, usability

## Abstract

**Aim:**

A clinical study was performed to test the usability of a smartphone eye-scanning app at a needle exchange facility to detect drug use to support therapy.

**Methods:**

The study recruited 24 subjects who visited the facility one to three times, making a total of 40 visits. During each visit the subjects underwent testing for non-convergence (NC), nystagmus (NY), and pupillary light reflex (PLR) using a smartphone-based eHealth system. The collected eye data were transformed into key features that represent eye characteristics. During each visit, a time-line follow-back interview on recent drug use and a usability questionnaire were completed.

**Results:**

Technical usability of the smartphone eye-scanning app was good for PLR and NC, where key features were generated in 82%–91% of the cases. For NY, only 60% succeeded due to cognitive problems to follow instructions. In most cases, subjects were under the influence of drugs when participating in the tests, with an average of 2.4 different drugs ingested within the last 24 h. The key features from PLR could distinguish use of opioids from central stimulants. The usability questionnaire results indicate that 23 of the 24 subjects could perform the eye-scanning by themselves after a short training, even when under severe influence of drugs. The caregiver assessed that 20 out of the 24 challenging subjects could potentially perform these tests in an indoors, home-like environment.

**Conclusions:**

Smartphone-based eye-scanning is functional in a patient population with heavy drug use, also when under the influence of drugs. The use of central stimulants can be distinguished from the use of opioids.

## 1 Introduction

“Everywhere, Everything, Everyone.” This is the year 2023 summary of the drug situation in Europe as assessed by the European Monitoring Centre for Drugs and Drug Addiction (EMCDDA) (1). The European drug situation is visible in many complex facets, such as homelessness, the management of psychiatric disorders, and youth criminality. A greater level of violence and corruption driven by the drug market is observed in some countries. Aligned with the EMCDDA report, the National Institute on Drug Abuse (NIDA) concludes that every dollar invested in addiction treatment programs results in a return of 4–7 dollars in reduced drug-related crime, legal costs, and theft (2). When healthcare savings are added, a return of at least 12 dollars is expected. Hence, research on the drug situation and methods to mitigate its detrimental powers is a strong need in Europe, the USA, and most probably worldwide.

The complex challenge of addressing contemporary drug-related problems has resulted in countless actions and research efforts, so far with small or hardly any practical effect on stopping drug overdosing. Following a widespread citation on overdose dynamics (3), recently published indicators on overdose management (4), drug-related crime (5), and societal cost (6) continue to be alarming, to mention only three examples. In fact, only 15–20 countries in the world have a GDP (7) greater than the estimated societal cost for managing opioid-use disorder and fatal opioid overdose in the US alone (6). Adding the COVID-19 pandemic to the picture reveals that irrespective of the healthcare organization, overdoses spiked during that time (8). Society suffers immensely, and the need for remedy is acute.

The drug testing arena is currently dominated by chemical tests of blood, urine, and saliva (9, 10). Each of these test modalities detects a chemical compound (the drug itself or a metabolite) and indicates use of drugs but not necessarily ongoing intoxication. This has led to complications in territories where cannabis is legal to use but not when driving (11). Also, designer drugs fall outside the scope of chemical tests because an existing test may not detect a newly developed designer drug rendering it difficult or impossible to conclude drug use through tests. An alternative could be to characterize the bodily reaction to drugs, to prove intoxication irrespective of cause.

Analysis of the eye for the purpose of indicating drug use has been discussed many times, and police handbooks often include tables with characteristic changes in eye reaction due to a variety of drugs (12). Although not conclusive and legally binding, such tables help law enforcement officers to understand if an individual could be under the influence of drugs and hence should be subjected to follow-up confirmatory tests. Attempts have been made to design technology that packages the ocular inspection of the police officer, to date with moderate success (13).

Amid the explosion of health apps available for virtually anything from general advice to specialized care, only some apps target the sector of substance-use disorder. An eHealth system for supporting treatment of alcohol abuse has been available for several years (14) and support for patients in opioid substitution therapy have been described (15), to name two examples. Digital smartphone tools have a unique edge in that they are widely used, fit into the pockets of patients, and are immediately available at any point in time. With adequate design, many patients can be managed with moderate resources. Considering the widespread problem of drug abuse, a digitalized solution should be seen as a key option for monitoring and managing patients with drug-use disorder.

In this study, we focus on a disregarded patient group: individuals with a severe drug addiction, and their ability to manage a smartphone-based drug monitoring app. Digitalization for such individuals is particularly challenging because the normal condition in this group is to be under the influence of drugs. The main question was to evaluate if it was at all possible for this patient group to use a smartphone to monitor drug use in a similar manner that is currently possible for alcohol-use disorder (14). Our hypothesis was that an eye-scanning app can be sufficiently robust and easy to use even for the most affected patient with substance-use disorder that is motivated to work toward sobriety.

## 2 Materials and methods

In accordance with the ethical permit, 24 subjects were recruited in a primary care facility dedicated to homeless individuals that includes a needle exchange station (*Mottagningen för särskild vård, Husläkarmottagningen för* *hemlösa och sprutbytet*, located in Uppsala, Sweden). The cohort characteristics are provided in Table 1. In total, 40 visits were made.

**Table 1:** Description of the study population, the number of visits performed, and device types.

Study population				
Age (years)	Subjects (*n* = 24)		Visits (*n* = 40)	Devices
Mean (standard deviation) Median (Min–Max)	Male	Female	Visits	Android iPhone
39.9 (11.5) 38.00 (18–64)	12	12	Visit 1 = 24 Visit 2 = 11 Visit 3 = 5	Samsung S22 = 11 iPhone 13 mini = 13

**Figure 1:**
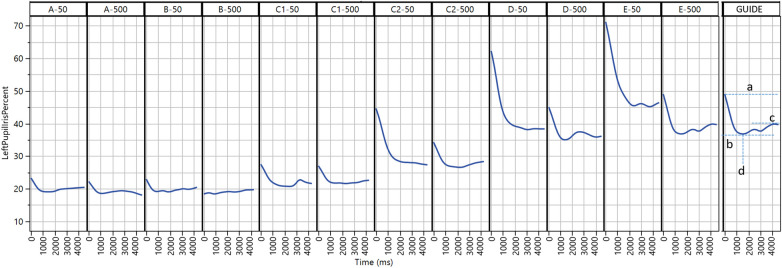
Collected data from the pupillary light reflex procedure from selected subjects (A–E) at two light conditions (∼50 and ∼500 lux). The final panel (GUIDE) illustrates extracted key features: Dbase (a), Dcon (b), Dend (c), and Ctime (d).

A smartphone-based eye-scanning app (Previct Drugs, Kontigo Care AB, Uppsala, Sweden) was used to collect data related to the eyes of the tested subjects. The tests were performed on two different smartphone types: Samsung S22 and iPhone 13 mini, even though the app works on most medium-priced smartphones where the camera resolution is 1,920 × 1,080 or better. Previct Drugs collects data from three procedures: pupillary light reflex (PLR), non-convergence (NC; also known as crossing-eye), and nystagmus (NY). When running the test, the app provided user guidance both using visual and “text-to-speech” generated audible instructions (16). After having video-filmed the eyes using the smartphone default settings (30 frames per seconds, auto exposure), each of the three procedures produced data that in turn was transformed into key features in the smartphone application. To produce a key feature, the sequence of pupil size over time (for PLR) and iris position over time (for NC and NY) were evaluated to extract characteristic features. Four key features related to PLR are schematically shown in Figure 1. PLR is the pupil response to transient illumination. Dbase represents the pupil size prior to illumination, Dcon represents the pupil size at maximum contraction due to illumination, Ctime is the time when Dcon occurs, and Dend is the pupil size at the end of the measurement.

The study took place at the needle exchange station. When someone arrived at the site, they were invited to participate. The site personnel gave written and spoken information about the study and the potential subjects were given time to read the information. Principal investigator (PI) or co-investigator answered all questions until the participants were adequately informed. The PI and co-investigator were regular staff at the site, meaning all subjects were familiar with them long before the start of the study and hence could discuss the study with ease. After written informed consent was obtained, the subject was considered as enrolled and the full eligibility process was performed. Thereafter, relevant medical/surgical history, review of relevant current medication, and information on demographics was collected.

To confirm drug use, self-reported data were collected using a time-line follow-back questionnaire at each visit. In addition, a Breath Explore (Munkplast AB, Uppsala, Sweden) breath air sampling device was used for voluntary testing, and the collected samples underwent high-pressure liquid chromatography/mass spectrometry/mass spectrometry (HPLC/MS/MS) analysis by Eurofins (Uppsala, Sweden) to chemically detect use of 36 drug compounds.

Each time a subject agreed to participate was denoted as a visit. During a visit, subjects were given a brief introduction (around 5 min) on how to use the app. They then conducted the eye-scanning without help in two different lighting conditions: NC, NY, and PLR tests at around 50 lux (dim indoor lights) and PLR test only around 500 lux (bright indoor lights), while the study staff at the same time recorded usability details in a questionnaire. In some cases, multiple measurements were made during a visit. After completing the measurement, subjects answered a short questionnaire on usability, and study staff recorded their observations. If a subject returned within a month of enrollment, a new measurement was performed, leading to multiple visits for some subjects (Table 1).

## 3 Results

The results from the usability questionnaire as answered by the caregiver while the subjects conducted eye-scans are summarized in Table 2. Of the 24 subjects that enrolled in the study, 19 managed to successfully complete the eye-measurements within one to two attempts. Regarding the data collected from the different eye-scanning tests, for 23 out of 24 subjects, the videos from PLR and NC tests could be converted into essential key features. However, in the case of the NY test, due to premature quality control handling in the app at the time of the study, only half of the tests produced key features. The caregiver assessed that for 4 out of the 24 subjects it was unlikely that they could perform the eye-scanning on their own.

**Table 2:** Results from the usability questionnaire related to 24 subjects, except for questions 5–7 (marked*) where data are related to 23 subjects.

Question					
	Good *N* (%)	Manageable *N* (%)	Less good *N* (%)		
1. Please evaluate the study subject's ability to use the mobile phone?	16 (66%)	7 (29%)	1 (4%)		
	Very good *N* (%)	Good *N* (%)	Either good or bad *N* (%)	Difficult *N* (%)	Very difficult *N* (%)
2. How did you perceive the study subject's ability to follow the instructions given by Previct Drugs?	5 (20%)	11 (45%)	6 (25%)	2 (8%)	0 (0%)
	Very easy *N* (%)	Easy *N* (%)	Either easy or difficult *N* (%)	Difficult *N* (%)	Very difficult *N* (%)
3. How did the study subject experience to put him/herself in the right position to be able to start a test?	2 (8%)	12 (50%)	3 (12%)	4 (16%)	3 (12%)
4. How would you evaluate the study subject's ability to perform a Cross-eyes test?	4 (16%)	9 (37%)	3 (12%)	6 (25%)	2 (8%)
5. How would you evaluate the study subject's ability to perform a Nystagmus test?*	10 (44%)	7 (30%)	3 (13%)	3 (13%)	0 (0%)
6. How would you evaluate the study subject's ability to perform a Contraction Test?*	5 (21%)	8 (35%)	2 (9%)	5 (21%)	3 (13%)
7. How did the study subject experience keeping the phone still during the test?*	3 (13%)	15 (65%)	2 (9%)	2 (9%)	1 (4%)
	Yes *N* (%)	No *N* (%)	Partly *N* (%)		
8. In addition to the basic instructions, did the study subject need additional support performing tests with Previct Drugs?	10 (41%)	14 (58%)	N/A		
9. Was the study subject able to perform the test after receiving additional support?	8 (33%)	1 (4%)	1 (4%)		
	1–2 times *N* (%)	3–4 times *N* (%)	More than 4 times *N* (%)		
10. How many efforts did it take the study subject to perform test with Previct Drugs?	19 (79%)	4 (16%)	1 (4%)		
	Very likely *N* (%)	Likely *N* (%)	Neither likely nor unlikely *N* (%)	Unlikely *N* (%)	Very unlikely *N* (%)
11. How likely do you think it is that the study subject will be able to perform tests with Previct Drugs without assistance in a home environment?	8 (33%)	8 (33%)	4 (16%)	3 (12%)	1 (4%)

The 40 visits produced a total of between 41 and 51 tests for the three test types (NC, NY, and PLR) at ∼50 lux and an additional 41 PLR tests at ∼500 lux. The NC test proved to be the most successful, with 91% of attempts (40 out of 44) producing eye-scanning data from which key features could be extracted. Conversely, the NY test was the least successful, with 40% of cases failing primarily due to subjects not following the instruction “keep head straight and look to the far left or right.” Instead, those who failed tended to turn their heads while looking to the far side. The overall results from the PLR test performed at both light conditions were 89% for PLR key features (82 out of 92).

Confirmed drug use was highly prevalent with an average of 2.4 different drugs ingested during the 24 h preceding a visit. Alcohol was reported 11 times, sleeping pills or benzodiazepines 12 times, cannabis 18 times, amphetamine 31 times, opioids 20 times, and other drugs 4 times. Drugs taken more than 24 h before the visit were disregarded. The voluntary breath tests as captured in a cartridge and analyzed with HPLC/MS/MS were, when present (in 33 of 40 visits), in line with time-line follow-back information for amphetamine.

**Figure 2:**
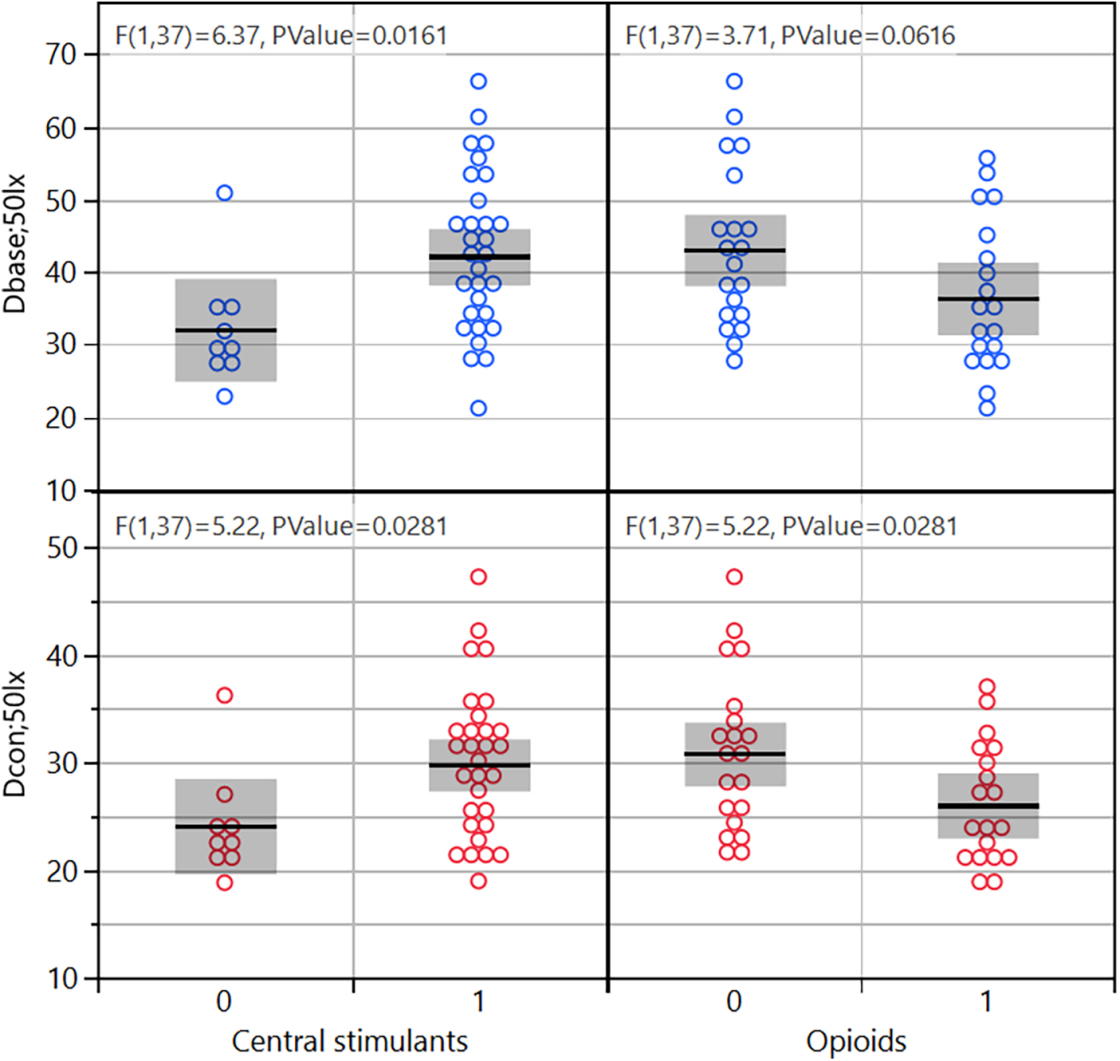
Two PLR key features (Dbase and Dcon) binned into central stimulants or not, and opioids or not.

Selected PLR key feature values collected at ∼50 lux are shown in Figure 2. Subjects with confirmed use of opiates in the past 24 h had smaller pupils and smaller contraction amplitude (*p* = 0.03–0.06). Subjects with confirmed use of amphetamine in the past 24 h had larger pupils (*p* = 0.02–0.03). The key features for the NC measurement did not correlate with drug use in this cohort. The key features for NY were not evaluated because of too large a proportion of missing data.

The most pronounced result indicating that a patient was under the influence of opioids (subjects A and B) was that they exhibited very low signals (Dbase ≈ 20, Dcon ≈ 20) and minimal responsiveness to the illumination of the torch (Figure 1, A and B). Subject A reported recent use of heroin. Subject B reported the use of both amphetamines and opioids within the last 24 h and exhibited no light reaction at ∼500 lux. Interestingly, subject B orally administered 1 g of tramadol and took two tablets of alprazolam at 22:00 the evening before the visit. Subject B also smoked cannabis and ingested 0.5 g amphetamine in the morning at 7:00 before a visit to the study clinic at 10:30. Since tramadol has a half-life of 8 h, the consumption of an extremely high dose 12 h before the visit resulted in a remarkably strong measurable opioid effect. This indicates that, in this instance, the significant dose of opioids taken the previous evening had a more pronounced impact than the use of amphetamine in the morning.

Subject C visited the study center three times, of which two are shown (Figure 1, C1 and C2), and displayed significant variations in collected data. During the initial visit (C1), the subject reported recent heroin and cannabis use. This is corroborated by the small initial pupil size under both lighting conditions (Dbase < 28), and light stimulation results in very small pupils (Dcon ≈ 20). During the second visit (C2), the subject reported a recent use of several drugs including amphetamine and had considerably larger pupils.

Subjects under the influence of amphetamine only (subjects D and E) displayed large pupils (Dbase ≈ 60–70) and large contraction minimum (Dcon ≈ 35–40) (Figure 1, D and E).

## 4 Discussion

Can a person under the influence of drugs conduct self-administered drug tests using a smartphone? The rightfully asked question now has an answer: Yes, most often so. By evaluating a smartphone-based eye-scanning app Previct Drugs among subjects recruited at a needle exchange program, one of the most challenging user groups was encountered with good results. This study also provides a glimpse into the reaction of eye physiology to real-world, high-dose drug use. It also proves that there is a chance to equip even the heaviest users with self-administered measurement technology to assist and support therapy.

Most of the subjects could conduct app-based drug testing after a short introduction, even when under the influence of heavy drugs such as heroin (Table 2). Two of the three data collection procedures (PLR and NC) worked fine in the hands of the subjects, whereas the NY test was misinterpreted by a too big fraction to be useful for analysis. Improved instructions and additional training are advised for the NY test. The confirmed capability of managing tests proves that distributed measurement programs are technically possible at all levels of addiction disease, because the subjects recruited to this study are among the heaviest drug users available. This illustrates that the heavy substance user has, in most cases, a high level of functionality both in motor function and in the ability to follow instructions.

The outcome, in terms of indicating drug use, could be evaluated for PLR and NC data. Although the confirmation of the fallout that opioids result in small pupils and amphetamines in large comes as no surprise, it is notable that these measurements were made with consumer hardware (a medium-priced smartphone) by the subject him/herself. Two types of studies dominated the past, on the one hand, the use of dedicated medical equipment to depict eye conditions in controlled conditions (17), and on the other, a more manual, in best cases semi-quantitative estimation of pupil size for legal purposes by Drug Recognition Experts (12). This study shows that quantification similar to the dedicated measurement situation with professional equipment can be obtained with much simpler electronics: An off-the-shelf medium-priced smartphone. Data collected for NC did not correlate with the drugs reported in the time-line follow-back reports. The exact performance of the smartphone-based eye-scanning cannot be estimated from this study but will be reported elsewhere using other data from an independent clinical study using the same device where healthy volunteers were subjected to pharmaceutical compositions in a controlled and safe manner (18).

Considering the global opioid epidemic, methods for distributed care in the rehabilitation and aftercare of painkiller addiction will likely grow in importance. The sheer volume of patients with painkiller addiction makes any attempt to traditional, visit-based care prone to failure, if not because of the cost, then because of a lack of therapists. Design of a treatment program that relies on home testing in a digitized, distributed manner would allow many more patients per therapist. Such a treatment program, if shown to be successful, stands a chance to tackle this epidemic. This study shows that distributed testing is possible from a usability aspect; the patients will be capable of self-administering eye-scanning-based drug tests in their home environment.

In the therapy for alcohol-use disorder, at least one eHealth solution has many years of follow-up data (14). The smartphone solution evaluated in this report is built on the same technology framework, which means that all peripheral aspects supporting the patient with the use disorder such as maintaining a diary, questionnaires on mood and motivation, communication, and the similar are readily available and combined with drug testing two to four times per day, all communicated directly to the therapist. Learnings from alcohol-use disorder indicate that patients motivated to stay sober often find the eHealth solution helpful in avoiding drinking. The same could be true for patients with substance-use disorder.

Management of both illicit and prescribed drugs among patients with substance-use disorder reaches far beyond the health of the individuals (6). Drugs are the root cause for entire sectors of organized crime and infect society with lethal violence among conflicting criminal organizations, too often with innocent citizens caught in the line of fire. By targeting the users and providing therapy, the demand for drugs could go down while simultaneously reducing crime. Hence, treating substance-use disorder on a systemic level should be highly prioritized.

The subjects that participated in the study deserve a special mention. Often considered to be at the lowest end of societal status, caught in a condition seen as shameful, and disregarded on most instances, these subjects in many cases were very knowledgeable and could teach practical drug pharmacology from own experience. The stigma surrounding substance-use disorder even made some of the authors of this report make estimates based on prejudice. No one thought that this study would recruit patients as easily as it did. No one thought that those enrolled in the study would engage as deeply as they did. While many of the study participants are so deep into abuse that their chances to recover and become clean are slim, their help to develop tools for those needing treatment in the future was genuine. We hope that we can match the effort of the study group and bring forward much better tools for treating substance-use disorder in the near future.

This study has several limitations. Since the study group was selected based on those who visited a needle exchange facility, the drug exposure was biased. The number of enrolled subjects was sufficient to establish usability data, but additional and complementary data must be collected to determine the performance of the method in terms of indicating drug use. The nature of the patient group is such that the order and schedule is difficult to maintain, hence data are unstructured and of *ad hoc* nature. This indicates that the statistical comparisons between drugs with balanced designs are not possible for this patient category. Neither is it possible to get a drug-free baseline as comparison. A parallel study on healthy subjects (18) was conducted to collect data for the development of detailed drug identification models. Even though it is known that different drugs affect the eyes in distinguished manners, an indirect measurement like eye-scanning introduces a risk for confounding drug use with any physiological or psychological state that impacts eyes. This is an advantage from a general therapy perspective, where co-abuse of drugs (19) may present a challenge in eye-scanning procedures if the effects of the multiple drugs used cancel the visible effects on the eyes, calling for frequent eye-scanning tests to minimize false negative results. This is however a disadvantage for cases where intoxication due to a specific drug is determined, such as by law enforcement.

In conclusion, an individual affected by heavy drug abuse will in most cases be capable of conducting self-administered smartphone-based drug testing. Since most individuals with a drug-use problem are less deeply into addiction than the evaluated patient group, the prospects for a digitalized, distributed drug monitoring system based on consumer grade hardware are excellent.

## Data Availability

The original contributions presented in the study are included in the article/Supplementary Material, further inquiries can be directed to the corresponding author.

## References

[1] EMCDDA. European Drug Report 2023: Trends and Developments. European Drug Report. (2023). 10.2810/161905

[2] NIDA. Principles of Drug Addiction Treatment: A Research-Based Guide. 3rd ed. NIH Publication No. 12–4180. (2018). Available online at: https://nida.nih.gov/sites/default/files/podat_1.pdf (Accessed February 28, 2024).

[3] Jalal H Buchanich JM Roberts MS Balmert LC Zhang K Burke DS. Changing dynamics of the drug overdose epidemic in the United States from 1979 through 2016. Science. (2018) 361(6408):eaau1184. 10.1126/science.aau1184 30237320 PMC8025225

[4] Skolnick P. Treatment of overdose in the synthetic opioid era. Pharmacol Ther. (2022) 233:108019. 10.1016/j.pharmthera.2021.108019 34637841

[5] Chen Q Sterner G Segel J Feng Z. Trends in opioid-related crime incidents and comparison with opioid overdose outcomes in the United States. Int J Drug Policy. (2022) 101:103555. 10.1016/j.drugpo.2021.103555 35026674

[6] Florence C Luo F Rice K. The economic burden of opioid use disorder and fatal opioid overdose in the United States, 2017. Drug Alcohol Depend. (2021) 218:108350. 10.1016/j.drugalcdep.2020.108350 33121867 PMC8091480

[7] Wikipedia. List of countries by GDP (nominal). Available online at: https://en.wikipedia.org/wiki/List_of_countries_by_GDP_(nominal).

[8] Friedman J Gjersing L. Increases in drug overdose deaths in Norway and the United States during the COVID-19 pandemic. Scand J Public Health. (2023) 51(1):53–7. 10.1177/14034948221075025 35120430 PMC9902899

[9] Allen KR. Screening for drugs of abuse: which matrix, oral fluid or urine? Ann Clin Biochem. (2011) 48(Pt 6):531–41. 10.1258/acb.2011.011116 21885472

[10] Verstraete AG. Detection times of drugs of abuse in blood, urine, and oral fluid. Ther Drug Monit. (2004) 26(2):200–5. 10.1097/00007691-200404000-00020 15228165

[11] DeGregorio MW Wurz GT Montoya E Kao CJA. A comprehensive breath test that confirms recent use of inhaled cannabis within the impairment window. Sci Rep. (2021) 11(1):22776. 10.1038/s41598-021-02137-x 34815467 PMC8611040

[12] Richman JE McAndrew KG Decker D Mullaney SC. An evaluation of pupil size standards used by police officers for detecting drug impairment. Optometry. (2004) 75(3):175–82. 10.1016/s1529-1839(04)70037-8 15058699

[13] Fazari GM. Testing the validity of pupillometer technology against traditional drug screening instruments. Fed Probat. (2011) 75(3). ISSN 0014-9128. https://www.uscourts.gov/sites/default/files/75_3_6_0.pdf

[14] Wallden M Dahlberg G Månflod J Knez R Winkvist M Zetterström A Evaluation of 6 years of eHealth data in the alcohol use disorder field indicates improved efficacy of care. Front Digit Health. (2024) 5:1282022. 10.3389/fdgth.2023.1282022 38250054 PMC10796677

[15] Hammerslag LR Mack A Chandler RK Fanucchi LC Feaster DJ LaRochelle MR Telemedicine buprenorphine initiation and retention in opioid use disorder treatment for Medicaid enrollees. JAMA Netw Open. (2023) 6(10):e2336914. 10.1001/jamanetworkopen.2023.36914 37851446 PMC10585416

[16] Hämäläinen M Zetterström A Andersson K. Quality assurance in medical facial images with respect to ambient illumination. Patent application WO2023249547. (2023).

[17] Monticelli FC Hitzl W Priemer F Preiss U Kunz SN Keller T. The potential of infrared pupillography in routine police traffic checks. Rechtsmedizin. (2015) 25:466–73. 10.1007/s00194-015-0023-8

[18] Clinical investigation KCClin01 NCT05731999. Available online at: https://classic.clinicaltrials.gov/ct2/show/NCT05731999 (Accessed February 28, 2024).

[19] Berndt S Rosenkranz M Martens MS Verthein U. Amphetamine-type stimulant dependence and association with concurrent use of cocaine, alcohol, and cannabis: a cross-sectional study. Eur Addict Res. (2022) 28(2):113–21. 10.1159/000519541 34788752

